# The effect of regular resistance exercise, vitamin D, and calcium supplements on the gastrocnemius muscle in rats in the post-menopausal period: An experimental study

**DOI:** 10.18502/ijrm.v19i3.8576

**Published:** 2021-03-21

**Authors:** Seyedeh Zahra Hosseini Sisi, Mohammad Ali Azarbayjani, Mohammadreza Vafaeenasab, Maghsoud Peeri, Mohammadreza Dehghani

**Affiliations:** ^1^Department of Exercise Physiology, Central Tehran Branch, Islamic Azad University, Tehran, Iran.; ^2^Yazd Cardiovascular Research Center, Shahid Sadoughi University of Medical Sciences, Yazd, Iran.; ^3^Yazd Medical Genetic Research Center, Shahid Sadoughi University of Medical Sciences, Yazd, Iran.

**Keywords:** Menopause, Muscle, Vitamin D, Calcium, Exercise.

## Abstract

**Background:**

Menopause is the natural termination of menstruation which affects the quality and important aspects of women's life.

**Objective:**

To evaluate the effect of regular resistance training (Ex) with vitamin D (Vit. D) and calcium (Ca) supplements in the postmenopausal period on muscle tissue in rats.

**Materials and Methods:**

In this experimental study, 72 female Wistar rats (8-10-wk old) were randomly divided into control, placebo, Vit. D, Ca, Ex, Ca + Vit. D, Ex + Ca, Ex + Vit. D, and Ex + Ca + Vit. D groups. Control and placebo groups were fed with a standard diet and sesame oil, respectively. Two month after the ovariectomy, Ex, Ca (35 mg/kg), and Vit. D (10000 IU) were administred in all groups except the control. The number of muscle and inflammatory cells, fiber diameter, endomysium thickness, and degenerative collagen fiber area were assessed through hematoxylin-eosin staining.

**Results:**

Muscle cell number was increased in the Ex + Vit. D + Ca, Vit. D + Ex, and Vit. D groups compared to the control group; also, inflammatory cell number showed significant increase in the Ex + Vit. D + Ca (12 ± 5.46), Vit. D + Ex (14 ± 3.25), Ex (13 ± 4.08), Vit. D (11 ± 3.26), Ca + Vit. D (10 ± 1.01), and Ca + Ex (9 ± 2.87) groups. Muscle fiber diameter in the Ex + Vit. D + Ca and Vit. D + Ex groups was higher than the other groups. Endomysium thickness was significantly decreased in the Ex + Vit. D + Ca and Vit. D + Ex groups compared to the control and placebo groups (p < 0.001). Degenerative collagen fiber area showed a significant increase in the Ex + Vit. D + Ca and Vit. D + Ex groups (p ≤ 0.001) comparison with the control group.

**Conclusion:**

Regular resistance exercise, Vit. D, and Ca supplements can improve muscle morphological features in the postmenopausal period.

## 1. Introduction

Women naturally spend a third of their lives in a stage called menopause (1). The average age of menopause in developed countries is 51 years, and approximately 4% of women experience menopause before the age of 40 (early menopause) (2). Physiologically, menopause is associated with a decrease in estrogen hormone secretion due to lack of follicular function and is characterized by symptoms such as exhaustion, depression, and muscle pain that have negative effects on quality of life (3).

Aging is associated with loss of muscle mass and muscle strength and function of the musculoskeletal system that is clinically known as sarcopenia (4). Although sarcopenia is characterized by loss and atrophy of muscle fibers, there is no identified scientific evidence for the cause of sarcopenia (5). Experiments have shown that postmenopausal women lose their muscle tissue rapidly within 10 years (6, 7). Studies have also shown that exercise (Ex) in old age can improve physiological conditions, increase protein biosynthesis, increase muscle strength and function, glycolytic fiber hypertrophy, and reduce or prevent the prevalence of sarcopenia (8, 9). Although physical activity has many benefits for postmenopausal women, studies available in this field are limited and have reported conflicting results. Aubertin-Leheudre and others, and Liberman and coworkers in two separate studies evaluated the effect of Ex on muscle size in the elderly (10). Although they did not show the effect of Ex on the muscle size in the postmenopausal period, they confirmed that there were evidences that Ex could improve muscle function.

Vitamin D (Vit. D), calcium (Ca), and parathormone metabolism are altered during menopause (11). Menopause is associated with changes in Ca metabolism. In recent years, bone mineral density test, has been able to show the sufficiency of Ca and serum Vit. D levels in human body (12). Gimigliano and colleagues found a positive relationship between Vit. D levels and muscle mass in the elderly. Also, they concluded that people with low Vit. D levels are at a higher risk of musculoskeletal disorders (6). Changes in skeletal muscles mass have significant negative effects on the quality of life. In addition, the burden of diseases increases with age, especially musculoskeletal disorders, so it is necessary to find a suitable method to preserve the strength of muscles in old age. Because women are more susceptible than men to age-related musculoskeletal disorders, it is important to investigate the factors of age-related musculoskeletal disorders in women. Since ex and good nutrition are two identified factors for the health of the musculoskeletal system, reports show that 81.3% of Iran's urban population has Vit. D deficiency and that this is higher in women than men (13, 14). Also, the prevalence of inactivity in women is higher in developing countries and this is associated with a high prevalence of musculoskeletal disorders (15).

Therefore, the present study aimed to investigate the effect of resistance Ex with Vit. D and Ca supplementations on the gastrocnemius muscle in rats in post-menopausal period.

## 2. Materials and Methods 

### Animal housing and treatment 

In this experimental study, 72 female Wistar rats (8-10-wk old, weighing 250 ± 15 gr) were randomly divided into control, placebo, Vit. D, Ca, Ca + Vit. D, Ex, Ex + Ca, Ex + Vit. D, and Ex + Ca + Vit. D groups. Animal house temperature (21 ± 2°C), relative humidity (30-40%), and 12-hr light-dark cycle were controlled and the rats were allowed free access to food and drinking water.

The different treatments were initiated 2 wk after the adaptation of animals. In all groups except the control and placebo groups, rats were ovariectomized and fed with a standard diet for two months. In order to make the ovariectomy, after the suture of the vascular plexus with fine linen thread, both ovaries were removed (16). Following the two months of housing, animals in the control group were fed with a standard diet until the end of the experiment, while those in the placebo group were only administrated sesame oil for 2 months. In the Ca group, Ca supplement (35 mg/kg, orally), and in the Vit. D group, intramuscular injection of 10000 IU/wk Vit D supplement were administered for 2 months in the Ca + Vit D group, both 35 mg/kg and 10000 IU Vit. D supplements were administrated for 2 months. In the Ex group, resistant Ex was performed for 2 months, additionally, in the Ex + Ca, Ex + Vit. D, and Ex + Ca + Vit. D groups, resistant Ex, Ca, and Vit. D were performed, respectively, during the two months. Resistance-trained rats were familiarized with climbing a ladder (1 m, 2 cm grid, 85 incline) before starting the resistance training. The initial weight was 30% of their body weight and was attached to their tails. Weights were increased to 100% of their body weight gradually throughout the 8 wk. The resistance training consisted of three sets of five repetitions with a 30-sec rest interval between the reps and 3 min between the sets (17). After the last resistance Ex sessions or the supplement administration, all animals were sacrificed by decapitation 24 wk after the beginning of the experiment (at 28 wk of age).

### Gastrocnemius muscle assessment 

Fresh gastrocnemius muscle was rinsed with PBS buffer, and then tissues fixed in a 10% formalin solution 5-10 times the volume of tissue for 24 hr at room temperature. After the fixation, the tissue samples were embedded in paraffin wax blocks as follows: one change 70% ethanol (30 min), 80% ethanol (2 hr), 95% ethanol (2 hr), three changes 100% ethanol (1.5 hr), two changes xylene (30 min). For embedding tissues into paraffin blocks, the melted hard wax was put into the embedding metal box, and then the tissue was placed quickly in the box. Paraffin blocks were trimmed as necessary and 5-µm slice thickness was commonly used. For rehydration, paraffin sections were placed in xylene for 10 min and in 100%, 95%, 80%, and 75% ethanol solution, each time for 3 min, and then rinsed with distilled water for 5 min. Slides were stained with hematoxylin and eosin (H&E) dyes for about 10 and 2 min, respectively. Eventually, the samples were put into 75%, 80%, 95%, l00% ethanol and xylene solutions for 5 min each (18). The microscopic check showed muscle tissue with the blue nucleus and red cytoplasm and fiber.

The morphological features of the muscle tissue such as the number of muscle nucleus, inflammatory and adipocyte area, fiber diameter, endomysium thickness, degenerative collagen fiber, fibrosis, the pattern of muscle fiber and interstitial edema were identified using a bright-light microscope (Olympus BX51, Japan, 200× magnification). Intact muscle tissue shows a homogenous fiber size, polygonal shape, and normal peripheral nuclei. Degenerating fibers have an irregular shape, few nuclei, and lighter fiber compared to normal muscle tissue. Fibrotic tissue and adipose tissue contain a high amount of light pink-to-whitish collagens and a large colorless and empty space among collagen structures, respectively. Expanded interstitial tissue with pale eosinophilic material that separates and surrounds individual myofibers shows edema in skeletal muscle (19).

### Ethical considerations

All protocols were performed according to the National Institute of Health Guidelines for the Care and Use of Laboratory Animals (NIH Publications No. 8023, revised 1978). The study was approved by the Animal Ethic Committee of Yazd Reproduction Sciences Institute, Shahid Sadoughi University of Medical Sciences, Yazd, Iran (IR.SSU.RSI.REC.1398.020).

### Statistical analysis 

The results of the experiments were expressed as mean and standard deviations of the different variables. The statistical analysis was done initially by SPSS, version 20 and one-way test. P < 0.05 and p < 0.001 were accepted as denoting a significant difference.

## 3. Results

Table I shows the muscle cell number, inflammatory cell number, and adipocyte cell area in the cross-section of gastrocnemius muscle. Clearly, the muscle cell number showed a significant increase in the Ex + Vit. D + Ca, Vit. D + Ex, Ex, and Vit. D groups (p ≤ 0.001), and the Ca + Ex and Ca + Vit. D groups (p = 0.04) in comparison with the control and placebo groups. However, the inflammatory cell number showed a significant increase in the Ex + Vit. D + Ca, Vit. D + Ex, Ex, Vit. D, Ca + Vit. D, and Ca + Ex groups compared to the control and placebo groups, no inflammatory cells were seen in the Ca group (p = 0.29). Additionally, the adipocyte cell area was significantly decreased in the Ex + Vit. D + Ca, Vit. D + Ex, Ex, Vit. D (p ≤ 0.001), Ca + Vit. D, and Ca + Ex (p ≤ 0.001) groups compared to the control and placebo groups.

**Figure 1:**
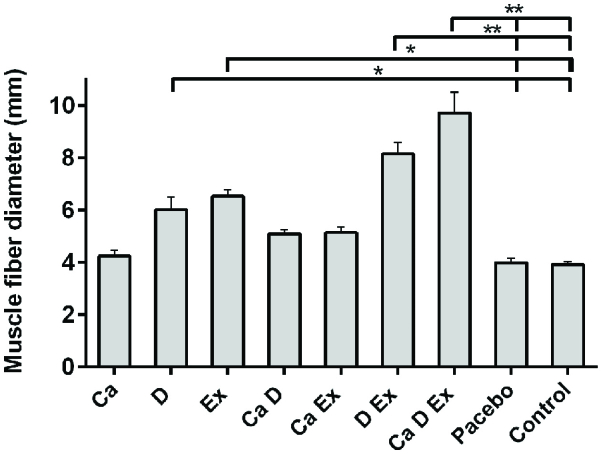
Comparison of muscle fibers' diameter of gastrocnemius muscle after resistant exercise and administration of calcium and Vitamin D supplements on ovariectomized rats among groups. *P ≤ 0.05 and **P ≤ 0.001 when compared to the control group. The statistical analysis was done initially by SPSS software, version 20 and one-way test. Ex: Resistant exercise; Ca: Calcium supplement; D: Vitamin D supplement.

**Figure 2:**
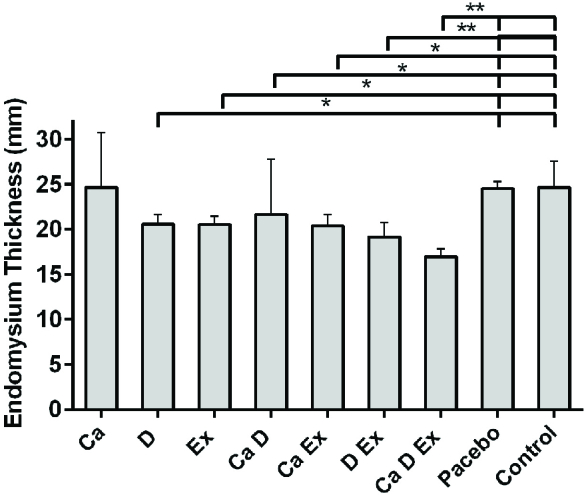
Comparison of endomysium thickness of gastrocnemius muscle in ovariectomized rats among groups. *P ≤ 0.05 and **P ≤ 0.001 when compared to the control group. The statistical analysis was done initially by SPSS software, version 20 and one-way test. Ex: Resistant exercise, Ca: Calcium supplement, Vit D: Vitamin D supplement.

**Figure 3:**
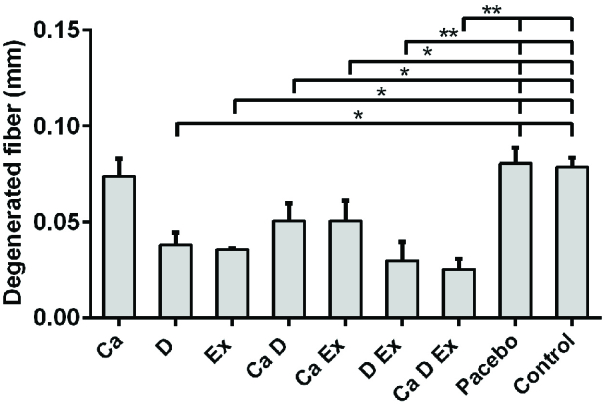
Comparison of degenerated fiber area of gastrocnemius muscle in ovariectomized rats among groups. *P ≤ 0.05 and **P ≤ 0.001 when compared to the control group. The statistical analysis was done initially by SPSS software, version 20 and one-way test. Ex: Resistant exercise, Ca: Calcium supplement, D: Vitamin D supplement.

**Figure 4:**
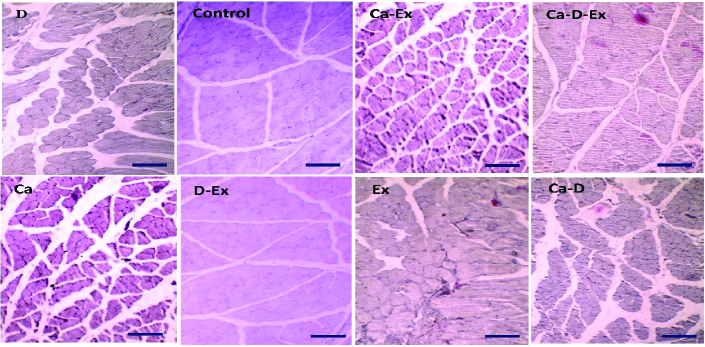
The pattern of gastrocnemius muscle fiber after resistant exercise and administration of calcium and vitamin D supplements in ovariectomized rats in all groups. 200× Magnification (Scale bars = 200 μm). Ex: Resistant exercise, Ca: Calcium supplement, D: Vitamin D supplement.

While a higher muscle fiber diameter was observed in the Ex + Vit. D + Ca and Vit. D + Ex groups (p < 0.001), a significant increase in the fiber diameter was seen in the Ex and Vit. D groups compared with the control and placebo groups (p = 0.02; Figure 1). Figure 2 shows that the endomysium thickness was significantly decreased in the Ex + Vit. D + Ca and Vit. D + Ex groups compared to the control and placebo groups (p < 0.001). However, there was a decreasing trend in the endomysium thickness in the Vit. D, Ex, Ca + Vit. D, and Ca + Ex groups (p = 0.01) among the control and placebo groups; however, no changes were seen in the endomysium thickness in the Ca group. Degenerative collagen fiber area showed a significant increase in the Ex + Vit. D + Ca and Vit. D + Ex groups (p < 0.001) and Ex + Ca, Ca + Vit. D, Ex, and Vit. D groups (p = 0.01) in comparison with the control and placebo groups (Figure 3). Morphological assessment of gastrocnemius muscle did not show change for fibrosis and interstitial edema among groups, also, there was no change in the pattern of muscle fiber (Figure 4).

**Table 1:** Comparison of the number of muscle cells, inflammatory cells, and adipocyte cells area after resistant exercise and administration of calcium and vitamin D supplements on ovariectomized rats


**Groups**	**Muscle cell number**	**Inflammatory cell number**	**Adipocyte cell area (mm)**
**Ca**	89 ± 6.09	3.13 ± 2	15.95 ± 4.32
**Vit. D**	156 ± 7.87**	11 ± 3.26**	9.62 ± 3.11**
**Ex**	143 ± 7.33**	13 ± 4.08**	8.93 ± 2.64**
**Ca + Vit. D**	105 ± 6.99*	10 ± 1.01*	11.66 ± 4.35*
**Ca + Ex**	109 ± 9.21*	9 ± 2.87*	13.35 ± 2.80*
**Vit. D + Ex**	153 ± 3.6**	14 ± 3.25**	7.38 ± 3.37**
**Ex + Vit. D + Ca**	162 ± 5.87**	12 ± 5.46**	6.45 ± 2.72**
**Placebo**	91 ± 11.03	0	17.00 ± 0.10
**Control**	91 ± 5.21	0	16.66 ± 4.09
**P-value**	≤ 0.001	≤ 0.001	≤ 0.001
Data presented as Mean ± SD. *P ≤ 0.05 and **P ≤ 0.001 when compared to the control group. The statistical analysis was done initially by SPSS software, version 20 and one-way test. Ca: Calcium supplement, Vit. D: Vitamin D supplement, Ex: Resistant exercise			

## 4. Discussion

Menopause is associated with a decrease in the estrogen level of women during the menopause period. This decrease in estrogen leads to loss of bone density, the decline in muscle mass, an increase in subcutaneous fat especially in the visceral area, increased risk of cardiovascular disease, and reduced quality of life (20). Also, decreased estrogen may have a direct effect on muscle tissue (21) so in this study we assessed the effect of regular resistance training in combination of Vit. D and Ca supplements on the muscle tissue in rats in their postmenopausal period.

The results of the present study in muscle cell number counting showed a significant increase in the Ex + Vit. D + Ca, Vit D + Ex, Ex, Vit. D, Ca + Ex, and Ca + Vit. D groups in comparison with the control and placebo groups. Maltais and co-workers reported a decline in muscle mass and strength after menopause and showed loss of muscle strength is associated with menopause (22). Muscles in the human body play an important role in physical function and quality of life. The Ex, good nutrition, hormone therapy, and Vit. D intake are essential factors to preserve muscle mass, strength, and quality of life. Fu and colleagues, similar to our result, reported that the muscle cells in women who practiced regular Ex and took Vit. D supplements for 12 wk repaired and the obesity decreased (23). Low physical activity, low protein intake, and high oxidative stress are the most common causes of sarcopenia in postmenopausal women (4). Evidence have shown the presence of estrogen receptors in human muscles (24). Dehaini and others reported that estrogen receptors are present in muscle cells, especially in type II muscle (25). These receptors may help to increase muscle cells after regular resistance Ex in premenopausal women. Perhaps, the increase in muscle cell number in Ex and supplement-intake groups in our study is due to the increase in estrogen receptors in muscle tissue.

It has also been suggested that Vit. D and regular Ex may also have positive effects on myogenic factors and subsequent proliferation of muscle cells and muscle growth due to decreased myostatin expression (a negative regulator of muscle growth) (26, 27). Based on this hypothesis, it seems that one of the most important causes of atrophy and a decrease in the number of muscle cells during the aging process is due to increased myostatin expression. Myostatin is a type of beta-modified growth factor, which is known to negatively regulate the process of myogenesis in skeletal muscle (28). Latres and colleagues in their animal experiments have shown that in myostatin gene expression-knocked animals, the muscle cells and muscle mass increased significantly. On the other hand, hypertrophy and severe hyperplasia formed in the muscle cell and fibers (29). Kim and co-workers showed after a resistance activity session, myostatin mRNA levels were significantly reduced in people aged between 50 and 75 yr (44%) (30, 31). Many reports suggested that Vit. D plays an important role in all body tissues, including the skeletal muscle, so that there are high levels of Vit. D receptors on the surface of skeletal muscle cells, and the number of these receptors decreased with aging (30, 31). Maybe, a decrease in myostatin level in our study groups (Ex and Vit. D) is responsible for increased muscle cells.

Our results in the inflammatory cell number showed a significant increase in the Ex + Vit. D + Ca (12 ± 5.46), Vit. D + Ex (14 ± 3.25), Ex (13 ± 4.08), Vit. D (11 ± 3.26), Ca + Vit. D (10 ± 1.01), and Ca + Ex (9 ± 2.87) groups over the control and placebo groups. Phillips and others in their study evaluated the inflammatory markers in menopausal women after Ex. They reported that Ex can elevate the inflammation in all tissues such as muscle and adipose tissue (32). Our result was similar to the Phillips' study. However, in contrast to our findings, Sirola and co-workers reported that training in menopause women can decrease inflammatory cells in muscles (33). A study by Grumati and colleagues assessed the effect of Ex on muscles in menopausal women. They reported that inflammatory calls elevated and muscle atrophy developed in a menopausal woman. Ex for the first time can increase inflammatory cells but when this Ex was regular and continued, the number of inflammatory cells decreased and muscular damage reduced (34). So, maybe the increase in inflammatory cells in our study was due to resistance Ex for 8 wk. Perhaps, a continuous Ex with supplementation in our study groups can decrease inflammatory cells.

The result of adipocyte cell areas in our study showed that the adipocyte cell area was significantly decreased in the Ex + Vit. D + Ca, Vit. D + Ex, Ex, Vit. D, Ca + Vit. D, and Ca + Ex groups compared to the control and placebo groups. Mason and others reported that in menopausal women, the level of fat and adipocyte cells are higher than that in a young woman. The reduction of sex hormone secretion is the main factor for aggregation of fat in all body such as adipocyte cells muscle cells (35). Ortmeyer and colleagues reported six-month Ex and healthy diets can decrease adipocyte cells in muscles (36). In our study, the Ex and Vit. D and Ca supplementations decreased the adipocyte cells in gastrocnemius muscle. Our findings were similar to Ortmeyer's results.

Joseph and co-workers reported that 12 wk of Ex cannot change the lipid profile and adipocyte cells in the menopause women (37). This was in contrast to our results. Maybe the difference between our result and Joseph's was the duration of training and supplementation with Vit. D and Ca in our groups.

The results of our study showed a fiber diameter increase in the Ex + Vit. D + Ca and Vit. D + Ex groups. This increase was significant in the Ex and Vit. D group compared with the control and placebo groups.

Studies have shown that in the young athletes, the muscle fibers' diameter was greater than the control group; however, limited data are available to assess the effect of Ex on the myofibrillar size in the elderly (38, 39). Skeletal muscle fibers have the ability to change the phenotype in response to environmental stimulation. This capacity is for adaptive change or cellular plasticity and hypertrophy after Ex. There is a general agreement that resistance Ex can cause hypertrophy in all types of muscle fibers. On the other hand, it is suggested that resistance Ex is an effective way to improve age-related atrophy and increase the size and strength of skeletal muscles in the elderly (40, 41). Consitt and colleagues in their study assessed the effect of resistance Ex on muscle cells. They reported after a period of resistance Ex in post-menopausal women, the level of androgens elevated and this elevation is responsible for an increase in muscle fiber diameters (42). Also, Maltais and others reported that resistance training and adequate protein intake can reduce the risk of sarcopenia and muscle loss. They suggested regular Ex and supplementation are beneficial to prevent menopausal complications. They have also reported that isoflavone supplementation could potentially be effective in increasing the secretion of sex hormones and has a constructive role in muscle mass in postmenopausal women (22). According to the results of the present study and other studies mentioned, Ex and Vit. D may be the two main factors for the production of androgens and thus increasing muscle fibers.

## 5. Conclusion

Our findings demonstrated that resistance Ex in combination with Vit. D represents preventive and therapeutic effect on the onset of muscle atrophy. Regular resistance Ex, Vit. D, and Ca supplements can improve muscle morphological features in the postmenopausal period. However, additional investigations are required for the evaluation of all effective parameters on muscle health, particularly from the molecular mechanism.

##  Conflict of Interest

The authors declare that there is no conflict of interest.
